# Supplementary material for: Motor expertise facilitates the accuracy of state extrapolation in perception
Source: PLoS One. 2017 Nov 6;12(11):e0187666. doi: 10.1371/journal.pone.0187666 (PMC5673241; doi:10.1371/journal.pone.0187666)
Supplement: S1 Appendix — (PDF) [file pone.0187666.s002.pdf]

S1 Appendix:
Motor expertise facilitates the accuracy of state extrapolation in perception

Nicolas Ludolph[1, 2]*, Jannis Plöger[1], Martin A. Giese[1], Winfried Ilg[1]

[1] Department of Cognitive Neurology, Section Computational Sensomotorics, Hertie Institute for Clinical Brain Research, and Centre for Integrative Neuroscience, University of Tübingen, Tübingen, Baden-Wuerttemberg, Germany
[2] International Max-Planck Research School for Cognitive and Systems Neuroscience, Tübingen, Baden-Wuerttemberg, Germany

* Corresponding author:

Email: nicolas.ludolph@uni-tuebingen.de (NL)

Cart-Pole balancing performance of motor familiar subjects

In order to assess whether the subjects in the group MF (N=9) are still able to balance the pole on the cart, we compared their performances in the motor control block, which precedes the last prediction block without feedback (T4) with subjects' end performances (Tend, N=30) reported in Ludolph et al. [1]. We did not find any significant difference between the two groups (p=0.78, Wilcoxon rank sum test), suggesting that subjects in the group MF were still able to balance the cart-pole system proficiently.

References

1. Ludolph N, Giese MA, Ilg W. Interacting Learning Processes During Skill Acquisition. Learning To Control With Gradually Changing System Dynamics. bioRxiv. 2017. doi: 10.1101/135095.
