# Supplementary material for: Motor expertise facilitates the accuracy of state extrapolation in perception
Source: PLoS One. 2017 Nov 6;12(11):e0187666. doi: 10.1371/journal.pone.0187666 (PMC5673241; doi:10.1371/journal.pone.0187666)
Supplement: S2 Appendix — (PDF) [file pone.0187666.s003.pdf]

S2 Appendix:
Motor expertise facilitates the accuracy of state extrapolation in perception

Nicolas Ludolph[1, 2]*, Jannis Plöger[1], Martin A. Giese[1], Winfried Ilg[1]

[1] Department of Cognitive Neurology, Section Computational Sensomotorics, Hertie Institute for Clinical Brain Research, and Centre for Integrative Neuroscience, University of Tübingen, Tübingen, Baden-Wuerttemberg, Germany
[2] International Max-Planck Research School for Cognitive and Systems Neuroscience, Tübingen, Baden-Wuerttemberg, Germany

* Corresponding author:

Email: nicolas.ludolph@uni-tuebingen.de (NL)

Comparison of visual information between the motor and visual groups

In order to verify that subjects in both groups were exposed to similar visual information during the familiarization blocks, we compared the kinematic information contained in the trials based on three features. Specifically, we compared sets of trials using the features: mean absolute pole angle (maPA), mean pole angle velocity (maPAV) and mean pole angle acceleration (maPAA). Since all subjects in the group VF watched the same familiarization stimuli ($N_{stimuli}$=100) but subjects in the group MF were exposed to their own, individual balancing attempts, we tested whether the set of familiarization stimuli (set_{VF}) is a likely sample from the set of all balancing attempts generated by the subjects in the group MF (N_{BA}=682). To this end, we first drew N_{sets}=10000 sets of size $N_{stimuli}$=100 from the set of all balancing attempts generated by the subjects in the group MF (bootstrapping). We then compared the stimulus set_{VF} to every drawn set by comparing the distributions of the three features using Kolmogorov-Smirnov tests. For each feature, we counted for how many of the drawn sets the stimulus set_{VF} is significantly different. We found that for 13.4% of the drawn sets at least one of the three tests revealed a significant difference, 2.4% at least two tests were significant and for none (0%) of the drawn sets all three tests were significant. This result strongly suggests that the set of presented stimuli is not significantly different from the sets drawn in the bootstrapping process. Thus, if we had first recorded all motor familiar subjects and then drawn from those a random set of balancing attempts for visual familiarization, the resulting set would not have been statistically distinguishable from the set we used. This also suggests that there is no difference in visual information (measured by the three features) during the familiarization blocks in the two conditions.
