# Supplementary material for: Motor expertise facilitates the accuracy of state extrapolation in perception
Source: PLoS One. 2017 Nov 6;12(11):e0187666. doi: 10.1371/journal.pone.0187666 (PMC5673241; doi:10.1371/journal.pone.0187666)
Supplement: S3 Appendix — (PDF) [file pone.0187666.s004.pdf]

S3 Appendix:
Motor expertise facilitates the accuracy of state extrapolation in perception

Nicolas Ludolph[1, 2]*, Jannis Plöger[1], Martin A. Giese[1], Winfried Ilg[1]

[1] Department of Cognitive Neurology, Section Computational Sensomotorics, Hertie Institute for Clinical Brain Research, and Centre for Integrative Neuroscience, University of Tübingen, Tübingen, Baden-Wuerttemberg, Germany
[2] International Max-Planck Research School for Cognitive and Systems Neuroscience, Tübingen, Baden-Wuerttemberg, Germany

* Corresponding author:

Email: nicolas.ludolph@uni-tuebingen.de (NL)

RESPONSE MODELS WITH LIMITED EXTRAPOLATION HORIZON

In order to investigate the hypothesis that subjects may only be able to predict the pole dynamics accurately over a limited period before having to switch to a heuristics, we investigated a class of partly heuristic forward models (lh_cVEL).

Let $t_{noi} = 4.5s$ be the time after which the force that is applied to the cart is clamped to zero and let $t_{occ} = t_{noi} + 0.1s = 4.6s$ be the time of the occlusion onset. Furthermore, let $t_{resp} = t_{occ} + 0.9s = 5.5s$ be the time when the subjects have to report their current pole angle estimate θ_{est}. For $t_{noi} \leq t \leq t_{resp}$, the true dynamics of the cart-pole system are described by the second-order differential equations (1) with the initial state $s_{init} = s(t = t_{noi}) = (x(t) \quad \dot{x}(t) \quad \theta(t) \quad \dot{\theta}(t))^T$.

$$\ddot{\theta} = F\big(\theta, \dot{\theta}, u = 0\big) = \frac{g \sin(\theta) + \cos(\theta) \left(\dfrac{-u - m_p\, l\, \dot{\theta}^2 \sin\theta}{m_c + m_p} \right)}{l \left(\dfrac{4}{3} - \dfrac{m_p \cos^2\theta}{m_c + m_p} \right)}$$

$$\ddot{x} = G\big(\theta, \dot{\theta}, \ddot{\theta}, u = 0\big) = \frac{u + m_p\, l \left(\dot{\theta}^2 \sin\theta - \ddot{\theta} \cos\theta \right)}{m_c + m_p} \tag{1}$$

These equations describe also the dynamics that are simulated by the model PERF, which consequently extrapolates the pole angle $\theta(t_{resp})$ without error. When assuming constant pole acceleration (cACC) or velocity (cVEL) after the occlusion occurred ($t \geq t_{occ}$), the estimated pole angle $\theta_{est}(t_{resp})$ is determined by simulating the dynamics, which are described by the differential equations in (2) and (3) with the initial state $s_{init} = s(t = t_{occ})$.

$$\ddot{\theta}_{est} = \ddot{\theta}(t_{occ}) \tag{2}$$

$$\dot{\theta}_{est} = \dot{\theta}(t_{occ}) \tag{3}$$

Our model lh_cVEL has one parameter h, $-100ms \leq h \leq 900ms$, which determines the duration of precise state extrapolation in milliseconds. It first either observes ($h \leq 0ms$) or extrapolates ($h > 0ms$) the state $s(t_{occ} + h)$ by simulating the true dynamics (equation 1) using the initial state $s_{init} = s(t = t_{occ})$. Then, it switches to the constant pole velocity model (cVEL) and simulates the corresponding dynamics (equation 4) for $t_{occ} + h \leq t \leq t_{resp}$ using the initial state $s_{init} = s(t = t_{occ} + h)$.

$$\dot{\theta}_{est} = \dot{\theta}(t_{occ} + h) \tag{4}$$

For $h = 900ms$ the model coincides with the model PERF, which represents perfect knowledge of the pole dynamics. Correspondingly, for $h = 0ms$ the model coincides with the model cVEL, which assumes that, the pole velocity remains constant over the time of the occlusion. We investigated 61 values of h corresponding to the 61 frames ($\Delta t = 1/60s$) from 100ms before ($h = -100ms$) to 900ms after ($h = 900ms$) the pole occlusion. The prediction error increases with increasing value of the parameter h. This corresponds to the decreasing accuracy of the model (**Figure 1**).

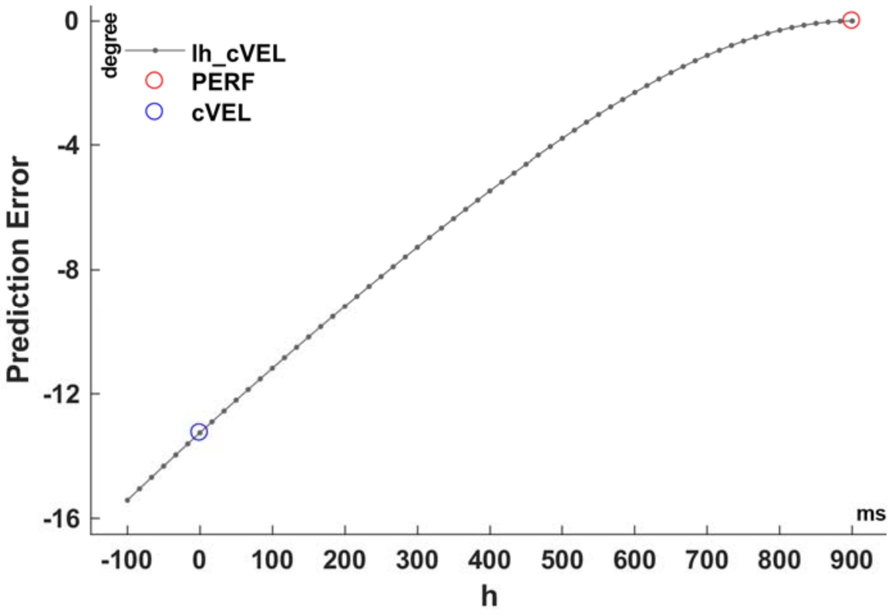

Figure 1. Prediction error as function of the extrapolation horizon (*h*) averaged over all stimuli. Notice that the prediction error monotonically increases with decreasing value of the parameter h.

In order to determine the best fitting model (h_s^*) for each subject (s), we first calculated the root mean squared error ($RMSE_{T4,s,h}$) between the models' ($MDL_h = \theta_{est}(t_{resp})$) and subjects' ($RSP_{T4,s}$) responses in block T4 for each model (h) (Equation S1).

$$RMSE_{T4,s,h} = \sqrt{\frac{1}{40}\sum_{trial=1}^{40}\left(RSP_{T4,s,trial} - MDL_{h,trial}\right)^2} \tag{S1}$$

The best fitting model (h_s^*) for each subject (s) was determined by minimizing the root mean squared error with respect to h (Equation S2).

$$h_s^* = \arg\min_h RMSE_{T4,s,h} \tag{S2}$$

Figure 2 shows the root mean squared error of all investigated models in the class lh_cVEL for two representative subjects, one from the group VF and the other from the group MF.

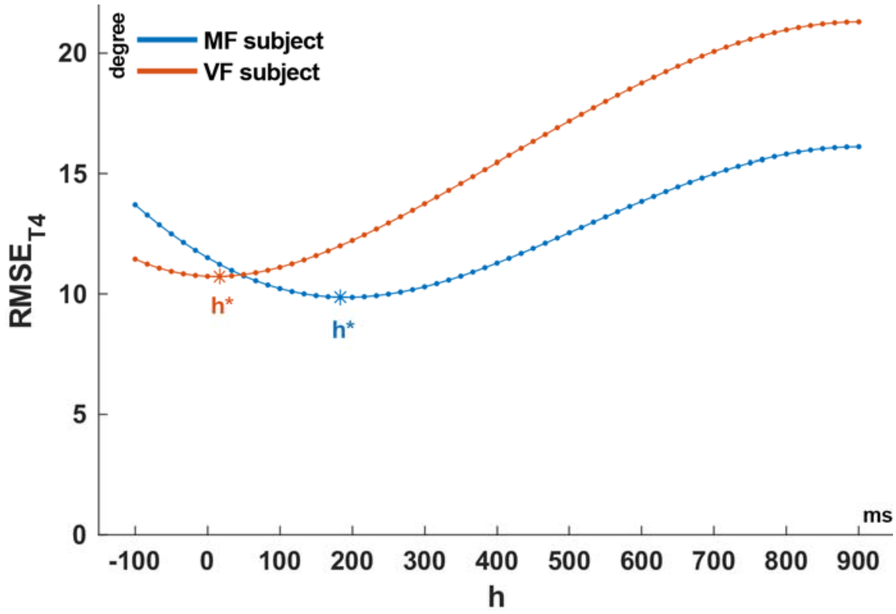

Figure 2. Root mean squared error of the model class lh_cVEL for two representative subjects. The best fitting model (h^{*}), according to the root mean squared error (RMSE) is for each of the two subjects indicated by a star. For the subject representing the group MF, h^{*} (extrapolation horizon) is higher (closer to the model which perfectly extrapolates the dynamic behavior of the pole: $h = 900ms$) than for the subject representing the group VF.
